# Restricted mean survival time of older adults with severe aortic stenosis referred for transcatheter aortic valve replacement

**DOI:** 10.1186/s12872-020-01572-4

**Published:** 2020-06-18

**Authors:** Julia Rodighiero, Nicolo Piazza, Giuseppe Martucci, Marco Spaziano, Kevin Lachapelle, Benoit de Varennes, Marie-Claude Ouimet, Jonathan Afilalo

**Affiliations:** 1 Research Institute of the McGill University Health Centre, Montreal, QC Canada; 2 Division of Cardiology, McGill University Health Centre, Montreal, QC Canada; 3 Division of Cardiac Surgery, McGill University Health Centre, Montreal, QC Canada; 4 Division of Cardiology and Centre for Clinical Epidemiology, Jewish General Hospital, McGill University, 3755 Cote Ste Catherine Road, Montreal, QC H3T 1E2 Canada

**Keywords:** Aortic stenosis, TAVR, Frailty, Survival

## Abstract

**Background:**

Few studies have measured frailty as a potential reason for foregoing transcatheter aortic valve replacement (TAVR) in older adults with severe aortic stenosis (AS). This study sought to determine the impact of frailty and other clinician-cited reasons on restricted mean survival time (RMST).

**Methods:**

An analysis of the McGill Frailty Registry was conducted between 2014 and 2018 at the McGill University Health Center Structural Valve Clinic. Consecutive nonsurgical patients referred for TAVR were included. In those that underwent balloon aortic valvuloplasty or medical management, the primary clinician-cited reason for foregoing TAVR was codified. Vital status was ascertained at 1 year and analysed using RMST and Kaplan-Meier analyses.

**Results:**

The study consisted of 373 patients with a mean age of 82.4 years, of which 233 underwent TAVR and 140 did not. Patients who did not undergo TAVR were more likely to be nonagenarians, with left ventricular dysfunction, chronic kidney disease, dementia, disability, depression, malnutrition, and frailty. The primary clinician-cited reason was: comorbidity in 34%, frailty in 23%, procedural feasibility and risks in 16%, and mild or unrelated symptoms in 27%. Compared to the TAVR group, 1-year RMST was reduced by 2.0 months in the medical management group (95% CI 1.2, 2.7) and by 1.1 months in the valvuloplasty group (95% CI -0.2, 2.5).

**Conclusions:**

Patients with severe AS referred for TAVR may never undergo the procedure on the basis of comorbidity, frailty, procedural issues, and symptoms. The best treatment decision is one that follows from multi-disciplinary assessment encompassing frailty.

## Background

Approximately 8% of individuals 80 years or older are affected by calcific aortic valve stenosis (AS), with the prevalence expected to grow in parallel to the aging population demographics [1]. Given the pathophysiological link between AS, aging, and cardiovascular risk factors, patients with AS often present at advanced ages suffering from multiple cardiac and non-cardiac comorbidities. In the past, one-third of patients were judged to be too old or comorbid to undergo surgical aortic valve replacement [2], and were therefore managed with palliative medical therapy. More recently, transcatheter aortic valve replacement (TAVR) has emerged as a less invasive option that was shown to be safe and superior to medical therapy for very high-risk older patients with severe symptomatic AS. The advent of TAVR has broadened the eligible patient population to include the oldest old who were previously not candidates. Even in nonagenarians, the procedural success rate has exceeded 95% and the 30-day and 1-year mortality rates have been estimated to be 5.5% and 23.0%, respectively [3]. However, despite these encouraging metrics, certain patients are evaluated and ultimately judged not to be good candidates for the TAVR procedure. The reasons may be multi-factorial owing to procedural considerations, comorbidities, and frailty. Few studies have systematically addressed the reasons for not proceeding with TAVR, and none have prospectively measured frailty using objective criteria as a key potential reason. Therefore, we sought to compare patients who proceeded with TAVR with those that did not to better understand the reasons cited by clinicians and patients for arriving at their decision and to determine the impact of these reasons on subsequent survival.

## Methods

### Study design

An analysis of the McGill Frailty Registry was conducted between 2014 and 2018 at the McGill University Health Center (Montreal, Quebec) clinic for structural heart valve disease. The Registry prospectively captures consecutive patients referred and evaluated for surgical or transcatheter heart procedures, including TAVR. In addition to the usual clinic assessment, a trained research assistant administers a comprehensive questionnaire and physical performance battery focused on frailty and other geriatric domains, and shares the results of these tests with the treating clinicians to help guide decision making. After the clinic visit and heart team discussion, a research assistant reviews the medical records to determine and codify the final treatment decision and justification, and they contact the patients to ascertain vital status at 1 year.

### Patient population

Inclusion criteria were (1) severe AS, (2) age ≥ 60 years, (3) signed informed consent to participate in the Registry, (4) clinical evaluation in the structural heart valve clinic for consideration of TAVR, (5) did not proceed with TAVR or cardiac surgery within 1-year of the index clinic visit. Patients who underwent TAVR during the study time frame were retained for comparative analyses. Patients who underwent balloon aortic valvuloplasty (BAV) were considered as part of the no TAVR group, unless they underwent subsequent TAVR within 1 year in which case, they were considered as part of the TAVR group. Exclusion criteria were (1) proceeded with surgical aortic valve replacement, and (2) referred for consideration of mitral, tricuspid, or pulmonic valve procedures.

### Clinical assessments

The clinic visit consisted of a multi-disciplinary assessment by a nurse practitioner, a structural cardiologist, and a research assistant from the FRAILTY team. The research assistant assessed the Essential Frailty Toolset (EFT) [4] for lower-extremity strength, cognitive function, albumin, hemoglobin, as well as assessments for upper-extremity strength, gait speed, balance, social support, depression, and disability for basic and instrumental activities of daily living. The patient underwent an electrocardiogram, echocardiogram, and if the clinician and patient were ready to consider TAVR, they underwent a computed tomography scan, coronary angiogram, and heart team discussion to integrate all of the information and arrive at a treatment decision.

### Reason for not proceeding with TAVR

When the decision not to proceed with TAVR was final, a research assistant ascertained the primary reason as documented by the treating clinician or the patient in cases when the latter refused intervention. These reasons were categorized as follows: (a) patient’s cardiac or extra-cardiac comorbidities, (b) patient’s frailty, (c) patient’s low symptom burden or symptoms not attributable to AS, (d) procedure’s technical feasibility or risks. In cases when more than one reason was cited, the predominant reason was chosen based on that which was most strongly or directly stated in the medical records. When uncertain, the research assistant asked the treating clinician to clarify.

### Statistical approach

Baseline characteristics and frailty traits were summarized with proportions, means and standard deviations and compared between TAVR and no TAVR groups using chi-squared tests and t-tests. One-year survival was compared between TAVR, no intervention, and BAV groups using (i) Kaplan-Meier analysis, (ii) Cox proportional hazards analysis adjusted for the Society of Thoracic Surgeons Predicted Risk of Mortality (STS-PROM) and the EFT score, and (iii) restricted mean survival time (RMST). The RMST indicates the average survival time over a pre-specified time frame, reflecting the differences in areas under Kaplan-Meier curves for patient groups. The RMST is not dependent on the proportional hazards assumption, and it reflects the entire time frame rather than instantaneous time points [5]. In the no TAVR group, 1-year survival was compared between subgroups of pre-defined reasons for not proceeding with TAVR. Statistical analyses were performed with STATA version 16 (College Station, TX).

## Results

### Baseline cohort characteristics

**Fig. 1:**
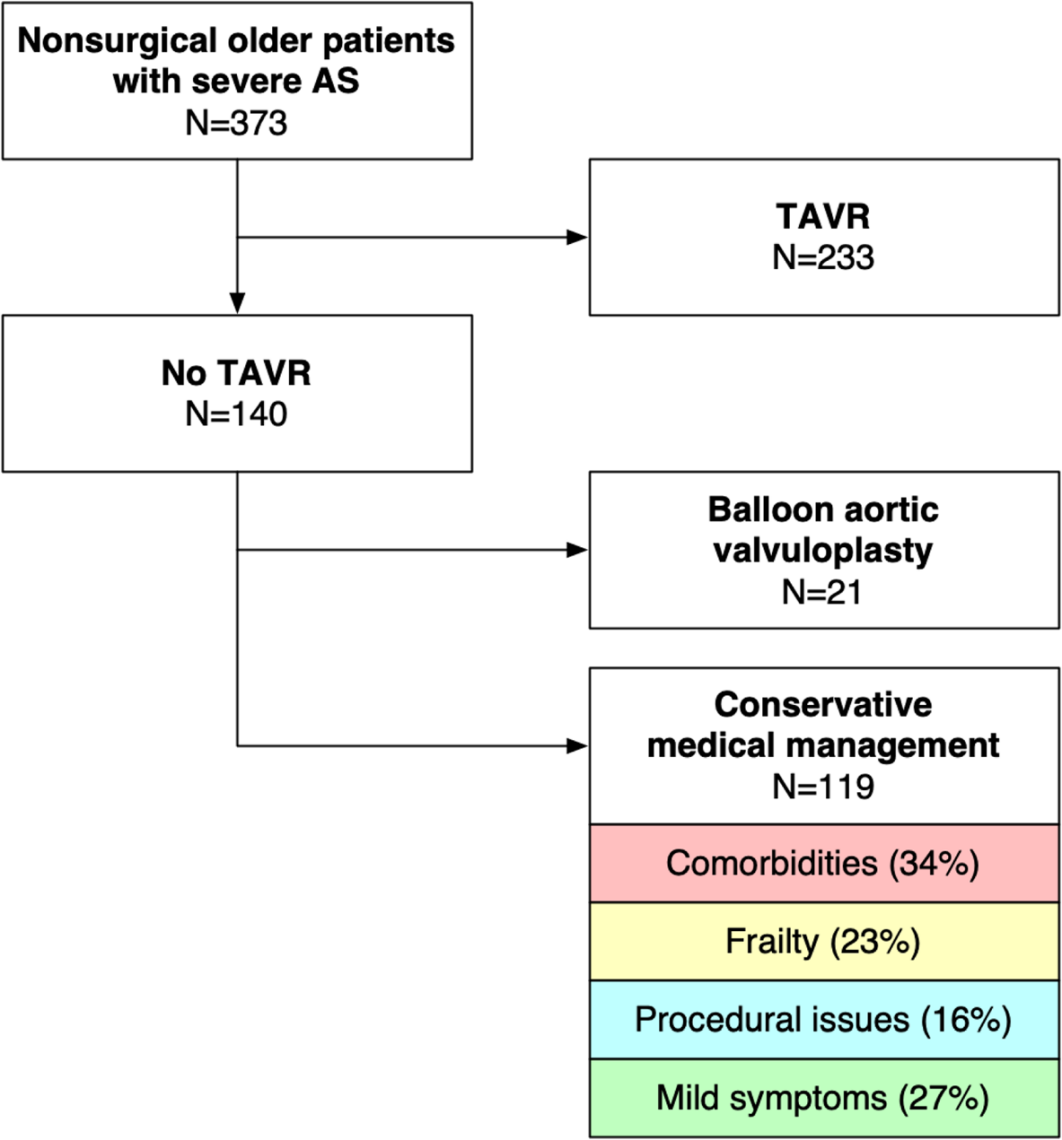
Flow Diagram. Abbreviations: AS, aortic stenosis; TAVR, transcatheter aortic valve replacement. Legend: A total of 373 older adults with severe AS were included: 233 underwent TAVR and 140 did not undergo TAVR. The primary cited reason for not undergoing TAVR was either comorbidities, frailty, procedural feasibility and risks, or mild or non-AS-related symptoms

The study consisted of 373 nonsurgical AS patients evaluated in the structural heart valve disease clinic between 2014 and 2018, of which 233 proceeded with TAVR and 140 did not (Fig. 1). Overall, the mean age was 82.4 ± 6.6 years and the mean STS-PROM was 4.4 ± 3.0%. Patients who did not proceed with TAVR had similar STS-PROM scores although they were older, more likely to be nonagenarians, more likely to have left ventricular dysfunction, chronic kidney disease, cognitive impairment, disability for activities of daily living, depression, malnutrition, and frailty according to various scales (Tables 1 and 2). The mean EFT score was 2.3 ± 1.1 vs. 1.6 ± 1.1 out of 5 in patients who did not proceed vs. proceeded with TAVR, respectively (*P* < 0.001).

**Table 1:** Clinical Characteristics

	Total (*N* = 373)	TAVR (*n* = 233)	No TAVR (*n* = 140)	*P*-value
DEMOGRAPHICS				
Age, years	82.4 ± 6.6	81.4 ± 6.3	84 ± 6.6	< 0.001
Female sex	184 (49%)	108 (46%)	76 (54%)	0.14
Body Mass Index, kg/m2	27.1 ± 6	27.4 ± 6.1	26.3 ± 5.7	0.12
COMORBIDITIES				
Diabetes mellitus	80 (21%)	66 (28%)	14 (10%)	< 0.001
Coronary artery disease	196 (53%)	137 (59%)	59 (42%)	0.002
Prior myocardial infarction	38 (10%)	26 (11%)	12 (9%)	0.42
Prior stroke	19 (5%)	17 (7%)	2 (1%)	0.01
Peripheral arterial disease	24 (6%)	19 (8%)	5 (4%)	0.08
Chronic kidney disease	187 (50%)	104 (45%)	83 (59%)	0.006
Dialysis-dependent	7 (2%)	5 (2%)	2 (2%)	0.85
Cirrhosis	7 (2%)	5 (2%)	2 (1%)	0.62
NYHA class	2.5 ± 0.6	2.4 ± 0.6	2.5 ± 0.7	0.15
ECHOCARDIOGRAM				
LVEF ≤35%	34 (9%)	15 (7%)	19 (14%)	0.02
PASP ≥60 mmHg	50 (13%)	27 (12%)	23 (16%)	0.18
Mean aortic gradient, mmHg	49.5 ± 18.3	52 ± 17.7	45.4 ± 18.6	< 0.001

Abbreviations: *LVEF* left ventricular ejection fraction, *NYHA* New York Heart Association, *PASP* pulmonary artery systolic pressure, *TAVR* transcatheter aortic valve replacement

**Table 2:** Geriatric Characteristics

	Total (*N* = 373)	TAVR (*n* = 233)	No TAVR (*n* = 140)	*P*-value
FRAILTY				
Essential Frailty Toolset	92 (26%)	43 (19%)	49 (40%)	< 0.001
Fried Frailty scale	99 (27%)	45 (19%)	54 (39%)	< 0.001
Rockwood Clinical Frailty Scale	98 (26%)	37 (16%)	61 (44%)	< 0.001
PHYSICAL DOMAINS				
Slow chair rise time	284 (76%)	158 (68%)	126 (91%)	< 0.001
Slow gait speed	215 (58%)	112 (48%)	103 (74%)	< 0.001
Weak grip strength	194 (52%)	103 (44%)	91 (65%)	< 0.001
Fall(s)	20 (5%)	13 (6%)	7 (5%)	0.81
NON-PHYSICAL DOMAINS				
ADL limitation	167 (45%)	79 (34%)	88 (63%)	< 0.001
Living in assisted facility	59 (16%)	33 (14%)	26 (19%)	0.26
Cognitive impairment	47 (13%)	18 (8%)	29 (21%)	< 0.001
Depression	88 (24%)	45 (19%)	43 (31%)	0.01
Malnourishment	32 (9%)	10 (4%)	22 (16%)	< 0.001

Abbreviations: *TAVR* transcatheter aortic valve replacement

Legend: Cut-offs for various domains were ≥ 3/5 for Essential Frailty Toolset, ≥3/5 for Fried Frailty Scale, ≥5/9 for Rockwood Clinical Frailty Scale, ≥15 s for slow chair rise time, ≥0.83 m/second for slow gait speed, < 30 kg in men and < 20 kg in women for weak grip strength, < 24/30 for Mini-Mental State Examination (cognitive impairment), ≥2/5 for Geriatric Depression Scale, < 8/14 for Mini-Nutritional Assessment

### Reasons for not proceeding with TAVR

Among 140 patients who did not proceed with TAVR, the primary cited reason was: comorbidity in 34%, frailty in 23%, procedural feasibility and risks in 16%, and mild or unrelated symptoms in 27%. When frailty was cited by the clinician as the primary reason, there was objective evidence of physical or psycho-social frailty in 94% of cases as measured by either the EFT, Clinical Frailty Scale, Fried scale, or disability for basic activities of daily living (with the remaining 6% being subjective impressions). Although comorbidity was often cited by the clinician, only chronic kidney disease was consistently predictive of no TAVR whereas many other comorbidities were more frequent in TAVR patients. A total of 16 patients died during the TAVR work-up period or while waiting for TAVR; most often patients who had been referred for additional testing or optimization of severe comorbidities before possible TAVR.

### Comparison of TAVR, BAV, and no intervention groups

**Fig. 2:**
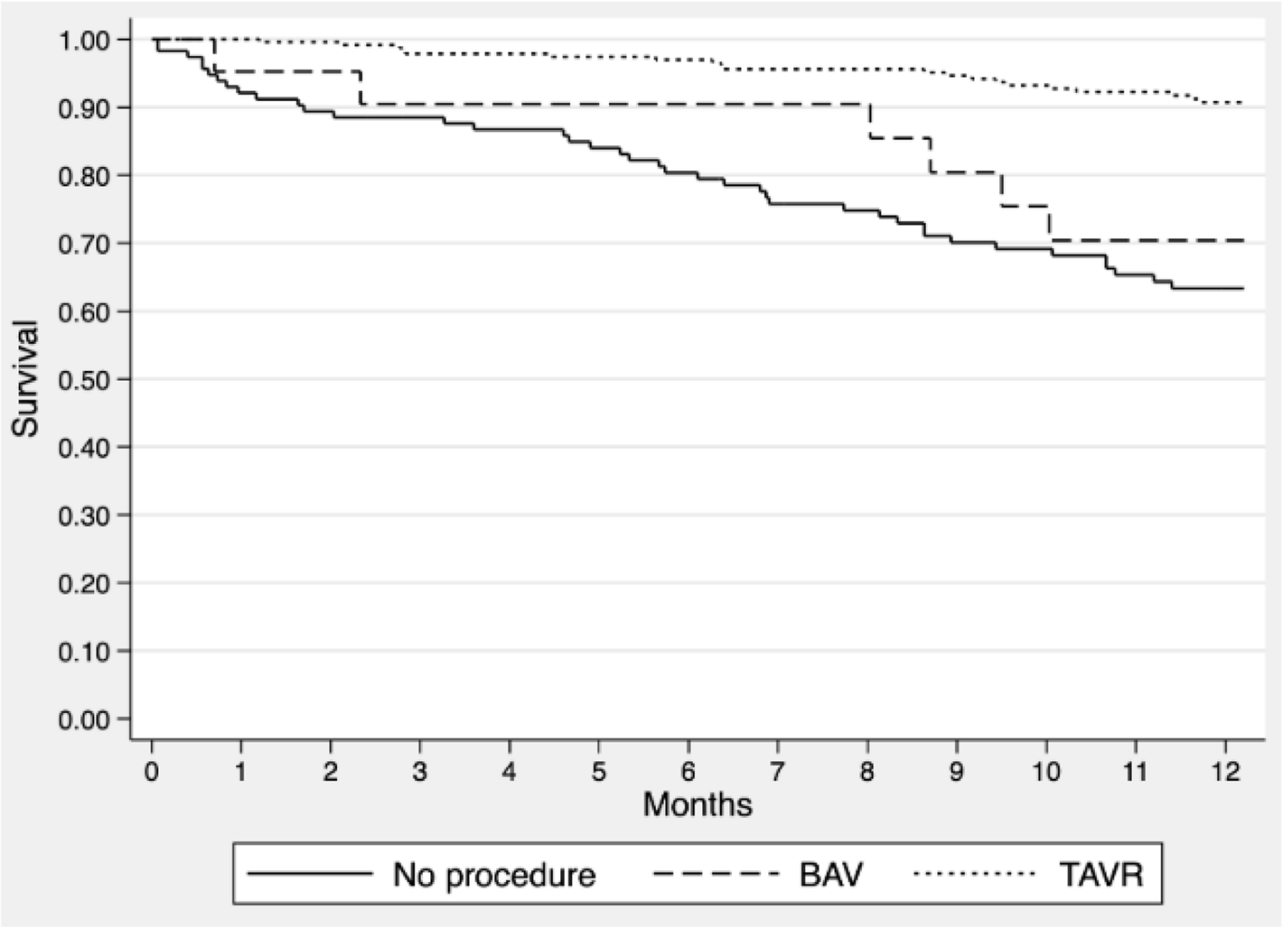
Survival by Treatment Group. Legend: Kaplan-Meier survival curves for patients referred to our clinic stratified by TAVR, BAV, and conservative medical management. Abbreviations as in Fig. 1

The Kaplan-Meier survival curves stratified by TAVR, BAV, and no intervention are shown in Fig. 2. RMST during the first year of follow-up was 11.6 months in the TAVR group (95% CI 11.3, 11.8), 10.4 months in the BAV group (95% CI 9.1, 11.7 months), and 9.6 months in the no intervention group (95% CI 8.8, 10.3). Compared to the TAVR group, RMST was reduced by 1.1 months in the BAV group (95% CI -0.2, 2.5) and by 2.0 months in the no intervention group (95% CI 1.2, 2.7). After adjusting for STS-PROM and EFT scores, there remained a trend towards reduced survival in the BAV group (HR 1.82; 95% CI 0.72, 4.60) and a significant reduction in the no intervention group (HR 4.59; 95% CI 2.61, 8.04). Inspection of the Kaplan-Meier curves showed that survival in the BAV group was relatively preserved up until 8 months post-procedure, after which time it declined to the level of the no intervention group.

### Comparison of reasons for not proceeding with TAVR

**Fig. 3:**
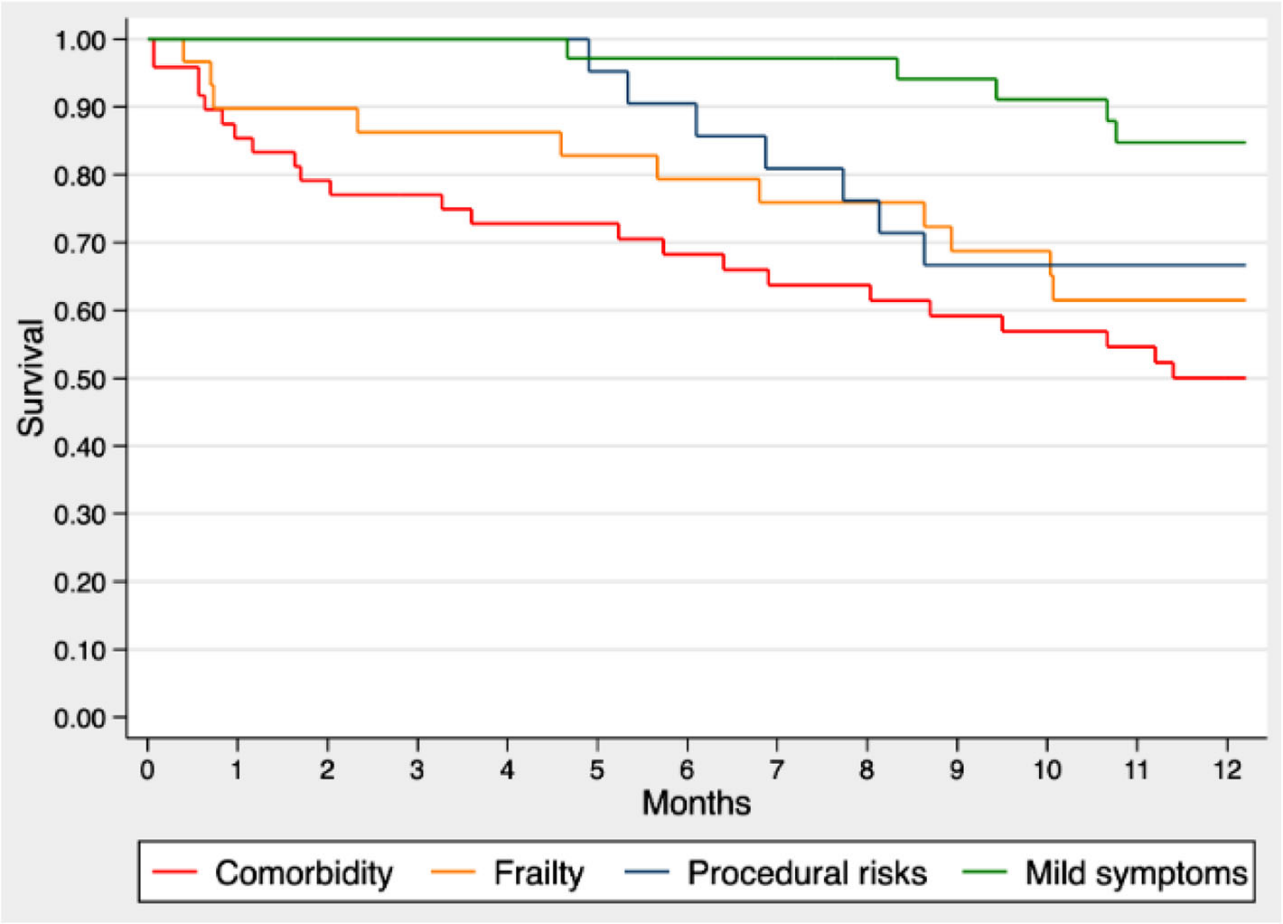
Survival by Clinician-Cited Reason for Foregoing TAVR. Legend: Kaplan-Meier survival curves for patients not undergoing TAVR, stratified by the clinician-cited primary reason for which TAVR was not performed. Abbreviations as in Fig. 1

The Kaplan-Meier survival curves stratified by comorbidity, frailty, procedural feasibility and risk, and mild or unrelated symptoms are shown in Fig. 3. RMST during the first year of follow-up was 8.3 months in the comorbidity category (95% CI 7.0, 9.6), 9.5 months in the frailty category (95% CI 7.9, 11.1 months), 10.3 months in the procedural feasibility and risk category (95% CI 9.0, 11.6 months), and 11.5 months in the mild or unrelated symptoms category (95% CI 10.8, 12.3). Compared to the mild or unrelated symptoms category, RMST was significantly reduced by 3.2 months in the comorbidity category (95% CI 1.7, 4.7) and by 2.0 months in the frailty category (95% CI 0.3, 3.8). Adjustment for STS-PROM and EFT scores was not performed since risk and frailty were embedded in the clinicians’ cited reasons.

## Discussion

This study from the McGill Frailty Registry examined the factors influencing clinical decision making and ensuing survival among older AS patients who received TAVR, BAV, or conservative medical management. The lessons learned can be summarized as follows. First, TAVR was associated with a gain in projected survival of 2 months within the first year as compared to conservative medical management. Second, BAV was associated with a projected survival similar to TAVR for approximately 8 months, after which time survival (in the absence of definitive TAVR) declined precipitously. Third, the primary reason for not proceeding with TAVR was cited as either comorbidity or frailty, and these patients had the highest mortality rates. Lastly, when the primary reason for not proceeding was mild or unrelated symptoms, these patients had relatively low mortality rates during the first year of follow-up.

Previous studies have described reasons for not proceeding with TAVR [6–8], however, these studies focused on comorbidities and symptoms and did not benefit from systematically-performed objective evaluations of frailty. This study further adds to the existing body of knowledge by connecting the various reasons for not proceeding with TAVR with projected survival. While comorbidity and frailty were associated with the lowest survival, deferral due to mild symptoms was associated with higher relative survival. Nevertheless, the 1-year mortality rate for mildly symptomatic patients was non-negligible, estimated at 15%, supporting the trend to perform aortic valve replacement sooner rather than later when AS is very severe – potentially even in asymptomatic patients [9].

An especially poor prognosis was observed when the cited reason for not proceeding with TAVR was comorbidities. Specific comorbidities found to be prevalent and influential for decision making were reduced LVEF, severe chronic kidney disease, and dementia; consistent with prior studies [10]. In a report from the STS/ACC TVT Registry, dialysis-dependent kidney disease was associated with a twofold increase in 1-year mortality rate up to 37% [11]. Other studies have reaffirmed the substantial increase in long-term and short-term risks [12, 13], including the procedural risk of contrast-induced acute kidney injury. In a meta-analysis, cirrhosis was also associated with a twofold increase in 1-year mortality [14]. In an analysis from the CoreValve Trial, oxygen-dependent lung disease was the comorbidity most strongly associated with 1-year mortality [15]. Overall, these data point to comorbidity profiles that may be equated with prohibitive risk or limited benefits to be expected from TAVR.

While frailty was commonly cited as a reason for choosing conservative medical management in our study and a previous study [16], frailty should not be equated with futility. It is important to note that one-half of TAVR patients manifest objective evidence of frailty at baseline, and that most still benefit from the procedure [17]. Those that typically do not benefit have severe frailty as defined by an EFT score of 5/5, advanced dementia, bedbound or non-mobile status, cachexia or severe sarcopenia, or disability for most basic activities of daily living. These risk factors, coupled with the aforementioned end-stage kidney, lung, and liver disease, have been grouped in the A-B-C-D-E mnemonic, which was put forth in the 2019 Canadian Cardiovascular Society TAVR Consensus Statement to guide decision making in potentially futile cases [18].

One of the strengths of this study is the use of the RMST to compare survival times in different subsets of patients who underwent or did not undergo TAVR. The RMST analysis yields results that are not only more intuitive to interpret, but are also more accurate for scenarios in which the hazard of an event changes over time [5, 19]. Cox proportional hazards analysis assumes that the hazard of an event is constant over the entire follow-up period, while logistic regression analysis assumes that the follow-up time period is constant and fixed for all patients; neither of which would have been ideal in this patient population. In the PARTNER B Trial, RMST was similarly used to demonstrate a gain in projected survival of 12.6 months within 5 years with TAVR as compared to conservative medical management [19].

BAV is used as a temporizing procedure to bridge the gap between TAVR and conservative medical management in challenging cases when patients suffer from severe comorbidities, frailty, or questionable symptoms. Epidemiologic and echocardiographic studies have shown the limited durability of the BAV procedure, with restenosis expected to occur at 6 months as evidenced by a mean aortic valve area progressing from 0.78 to 0.65 cm^2^ [20]. Once restenosis occurs, the long-term survival approaches the natural history of severe AS patients [20–22]; a finding which is supported by our data. Thus, our results and those of others suggest that patients should be reassessed at 6–8 months post-BAV to reconsider a more definitive procedure if clinically appropriate and in-line with the patient’s preferences.

### Limitations

Firstly, this was a single-centre study at an academic university hospital, therefore the reasons for not performing TAVR may not be generalizable to other centres with different practice patterns. Secondly, cause of death was not recorded in this study, although it would have been of interest to examine the distribution of cardiac and non-cardiac causes of deaths in our patient subgroups. Thirdly, time of follow-up was affected by immortal-time in the TAVR and BAV subgroups, since these patients necessarily survived to the time of their procedure while (a small number of) the remaining patients died early before any procedure could be performed. The likelihood of bias was minimized [23] by using the same time zero for all patients, which was defined as the date of the initial structural heart valve disease clinic visit. Fourthly, the RMST does not represent the true benefits of TAVR relative to BAV over a modest 1-year time period. The RMST is known to be skewed in scenarios with few early events [5]. Finally, this study was not designed to define indications for proceeding with TAVR or other treatment strategies; rather, to clearly understand the reasons and outcomes of patients not proceeding with this procedure.

## Conclusions

Patients with severe AS referred for TAVR may never undergo the procedure based on concerns surrounding comorbidity, frailty, procedural feasibility and risks, and mild or unrelated symptoms. These concerns – elicited by clinicians or patients – should be thoroughly investigated and discussed using a shared-decision making approach [24]. A comprehensive treatment decision is one that follows from multi-disciplinary assessment, including objective assessment of frailty and disability, which informs the expected risks and benefits in an individualized fashion. Structured decision aid tools have been developed and validated to facilitate this process [25].

## Data Availability

The datasets analysed during the current study may be made available from the PI on reasonable request.

## Abbreviations

TAVR: Transcatheter aortic valve replacement
AS: Aortic stenosis
RMST: Restricted mean survival time
EFT: Essential frailty toolset
STS-PROM: Society of Thoracic Surgeons Predicted Risk of mortality
BAV: Balloon aortic valvuloplasty
LVEF: Left ventricular ejection fraction
NYHA: New York heart association
PASP: Pulmonary artery systolic pressure
